# Role of δ-Aminolevulinic Acid Dehydratase (ALAD) gene polymorphism in lead induced nephrotoxicity

**DOI:** 10.1186/1755-8166-7-S1-P50

**Published:** 2014-01-21

**Authors:** Mugdha Tiwari, LJ Bhagia, I Shaikh, D Rohila

**Affiliations:** 1 Environmental Carcinogen Unit, National Institute of Occupational Health, Ahmedabad, India; 2 Hygiene Department, National Institute of Occupational Health, Meghani Nagar, Ahmedabad, India

## Background

Lead is an important environmental and occupational pollutant and it can cause nephrotoxicity even at low doses. Early detection of renal disease from occupational and environmental exposure to nephrotoxic chemicals is currently limited by the lack of sensitive or chemical specific tests. There are growing body of evidence that supports Kidney injury molecule-1 (KIM-1) as a specific marker for nephrotoxicity, specifically- ischemic renal injury. δ- aminolevulinic acid dehydratase (ALAD) gene polymorphism is known as an important factor affecting workers susceptibility to lead toxicity, further role of ALAD-1-1 and ALAD2-2 genotype is not clear yet. Correlation among the blood lead level, ALAD genotype present, urinary KIM-1 level of workers can provide better understanding about lead intoxication pattern and the role of genetic factor in susceptibility towards lead intoxication among workers.

## Methods

A total 200 biological samples (blood and urine) collected from workers working in lead-acid battery manufacturing unit, were analysed for blood lead levels, urinary KIM-1 and presence of ALAD genotype.

## Results

In the present study significant correlation between blood lead levels and kidney toxicity were found. In individuals having ALAD 1-1 genotype, blood lead levels were observed significantly higher.

## Conclusion

Higher KIM-1 level in urine in lead exposed workers denotes that KIM-1 can be considered as early kidney injury for lead induced nephrotoxicity.

